# Attempts to Improve Lipophilic Drugs’ Solubility and Bioavailability: A Focus on Fenretinide

**DOI:** 10.3390/pharmaceutics16050579

**Published:** 2024-04-24

**Authors:** Silvana Alfei, Guendalina Zuccari

**Affiliations:** 1 Department of Pharmacy, University of Genoa, Viale Cembrano 4, 16148 Genoa, Italy; silvana.alfei@unige.it; 2 Department of Pharmacy, University of Genoa, Viale Benedetto XV, 16132 Genoa, Italy

**Keywords:** retinol, natural retinoids, all-trans retinoic acid (ATRA), fenretinide (4-HPR), low bioavailability, 4-HPR oral formulations, 4-HPR parenteral formulations, lipophilic drug, preclinical and clinical trials

## Abstract

The development of numerous drugs is often arrested at clinical testing stages, due to their unfavorable biopharmaceutical characteristics. It is the case of fenretinide (4-HPR), a second-generation retinoid, that demonstrated promising in vitro cytotoxic activity against several cancer cell lines. Unfortunately, response rates in early clinical trials with 4-HPR did not confirm the in vitro findings, mainly due to the low bioavailability of the oral capsular formulation that was initially developed. Capsular 4-HPR provided variable and insufficient drug plasma levels attributable to the high hepatic first-pass effect and poor drug water solubility. To improve 4-HPR bioavailability, several approaches have been put forward and tested in preclinical and early-phase clinical trials, demonstrating generally improved plasma levels and minimal systemic toxicities, but also modest antitumor efficacy. The challenge is thus currently still far from being met. To redirect the diminished interest of pharmaceutical companies toward 4-HPR and promote its further clinical development, this manuscript reviewed the attempts made so far by researchers to enhance 4-HPR bioavailability. A comparison of the available data was performed, and future directions were proposed.

## 1. Introduction

Although there is a heightened awareness among the scientific community of the importance of the physicochemical properties of a drug candidate, currently more than 40% of drugs are water-insoluble, thus showing bad biopharmaceutical features [1]. Lipophilicity is one of the most important physicochemical descriptors and it can manage both pharmacodynamic and pharmacokinetic profiles of a drug, influencing its absorption, metabolism, distribution, excretion, and toxicity (ADMET). It particularly affects the ability of a molecule to cross membranes, bind to plasma proteins or receptors, accumulate in tissues, and be extensively metabolized [1]. Therefore, lipophilicity is generally considered the most important reference parameter for predicting the possible successful passage of a new drug from clinical development to the marketplace. Physically, lipophilicity is expressed as the logarithmic *n*-octanol-water partition coefficient (log P) and is characteristic of a given molecule. It has been proven that a log P value > 5 is associated with undesirable features, such as tissue accumulation, fast metabolic turnover, poor water solubility, or strong plasma protein binding. In particular, studies indicated that the log P values to achieve good bioavailability following oral administration should be in the range 0–3 [2].

**Figure 1:**
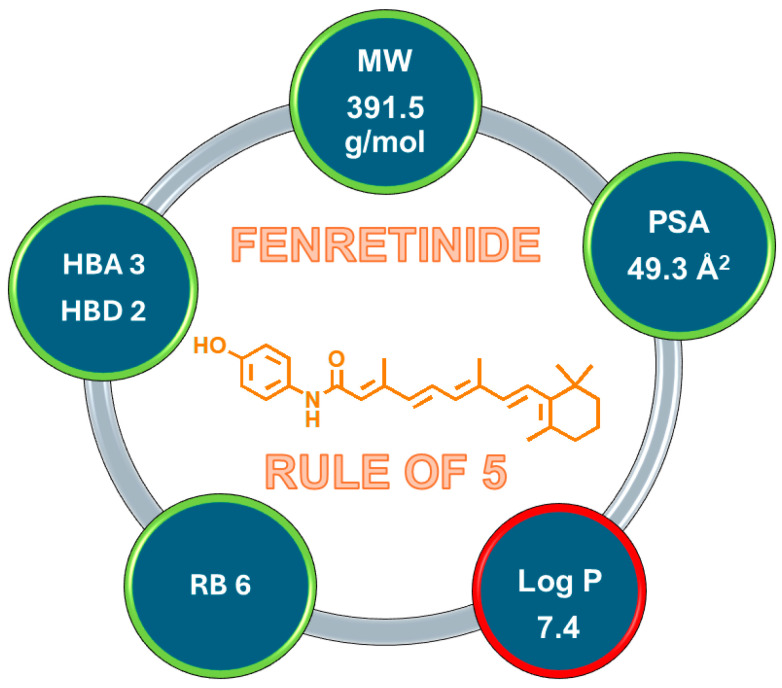
Application of the Rule of Five by Lipinski to 4-HPR. Molecular weight (MW), polar surface area (PSA), rotatable bonds (RB), hydrogen bond acceptors (HBA), hydrogen bond donors (HBD), log P. Circles with green outline fit the RO5 requirements, while the circle with red outline represent a RO5 violation.

It is well-known that, when given by the oral route, an ideal drug molecule would comply with the physicochemical property guidelines of Lipinski’s rule of five (RO5). According to the RO5, a drug-like compound should have a molecular weight of less than 500 g/mol, a log P < 5, no more than 5 hydrogen bond donors (HBD), and no more than 10 hydrogen bond acceptor (HBA) sites. Subsequently, two further conditions, correlated with drug permeability and flexibility, such as a polar surface area (PSA) < 140 Å and fewer than 10 rotatable bonds (RB), were added [2]. However, despite the existence of such guidelines to closely predict the ADME of orally administered drugs, most currently available anticancer drugs do not meet the RO5 requirements, simply because they are often given intravenously rather than orally or because they were developed before the RO5 was published. This is the case with fenretinide (4-HPR), synthesized in the late 1960s by Robert J. Gander at the Johnson & Johnson (J&J, Santa Clara, CA, USA) pharmaceutical company. The scope of this new synthetic molecule was to obtain a retinoic acid (ATRA) derivative to treat skin diseases with fewer adverse effects [3]. Unexpectedly, while 4-HPR was found to be inactive in dermatologic applications, it emerged as a synthetic retinoid with promising anticancer effects, as demonstrated by numerous in vitro studies [4,5]. 4-HPR’s physicochemical properties meet the RO5 requirements, except for the log P values, as visually reported in Figure 1. 

Thus, for this molecule, the main drawback is represented by its poor aqueous solubility (1.71 µg/mL) [6], which limits not only its dissolution rate in the biological fluids but also its absorption, since 4-HPR lipophilicity favors the permanence of the molecule inside the hydrophobic milieu of the membrane bilayer rather than its crossing. Due to these characteristics, 4-HPR has been included in Class IV (low solubility and low permeability) of the Biopharmaceutical Classification System (BCS) [7].

**Figure 2:**
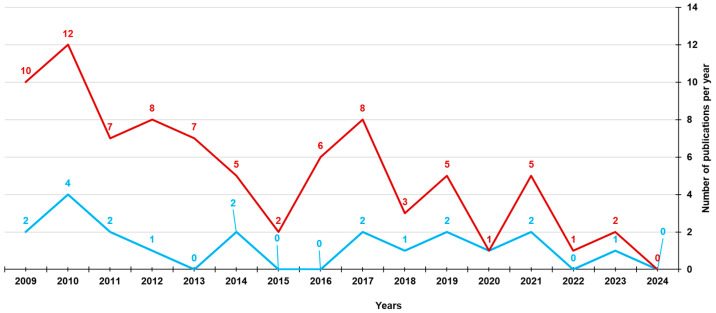
Number of articles published since 2009, including in vivo assessments of 4-HPR formulations according to PubMed dataset. The survey was carried out using the keywords “fenretinide” and “preclinical studies” (light blue line) and “fenretinide” and “clinical trials” (red line).

In the beginning, following promising results from in vitro investigations, 4-HPR was formulated in capsules for oral administration to perform clinical studies. A capsular oral formulation, made of soft gelatine capsules containing 4-HPR dissolved in corn oil and polysorbate 80, was available at the National Cancer Institute (NCI-FeR). Unfortunately, the antitumor effectiveness of capsular 4-HPR in clinical trials did not satisfy expectations, since this formulation required the administration of excessively elevated drug doses to achieve the plasma therapeutic concentrations that constrained drug tolerability and patient compliance [8]. Consequently, 4-HPR has not gained any approval so far and is currently unavailable on the market. This lack of commercialization is most likely due to therapeutic failures during clinical trials, linked to its strongly limited bioavailability. In the last decades, several efforts have been made by the scientific community to develop 4-HPR delivery systems with enhanced drug aqueous solubility and bioavailability, thus allowing plasma concentrations to reach levels sufficient to trigger a satisfactory therapeutic response. Meanwhile, the interest of the pharmaceutical companies in this molecule rapidly waned, due to the high costs of formulation development. In this review, to redirect the diminished interest of the pharmaceutical sector towards 4-HPR and promote its further clinical development, we first describe the chemical features of retinoids, to which 4-HPR belongs, then report the formulative approaches carried out to improve 4-HPR’s pharmacokinetic, bioavailability, and therapeutic efficacy in the treatment of cancer. Since the aim of this review was to summarize the different attempts made so far to increase the bioavailability of 4-HPR, only research works reporting preclinical or clinical studies in the field of cancer treatment have been considered. To this end, a search by the keywords “fenretinide” and “clinical trials” or “fenretinide” and “preclinical studies” was carried out on PubMed to identify suitable works. Figure 2 shows the results of our survey concerning the last fifteen years.

Figure 2 clearly demonstrates that the interest in 4-HPR has waned over time, going from 10 to 12 papers published per year in 2009–2010 (clinical trials) to only two published last year. Among these papers, according to our scope, we mainly considered those research works reporting data from pharmacokinetic studies and in vivo therapeutic efficacy evaluations in the field of cancer treatment.

## 2. Retinoids

**Figure 3:**
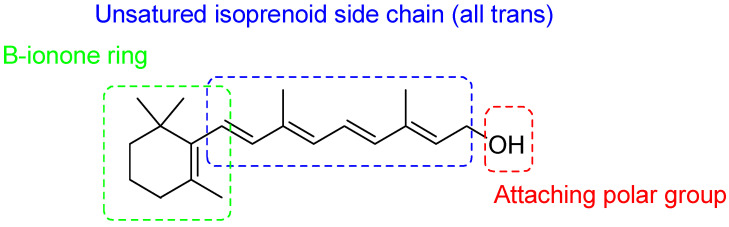
Chemical structure of retinol.

According to the IUPAC (International Union of Pure and Applied Chemistry) and IUBMB (International Union of Biochemistry and Molecular Biology), retinoids are compounds containing four isoprene units with a head-to-tail structure [9] that link a polar head to a non-aromatic fragment (β-ionone), as observable in the chemical structure of Vitamin A (retinol) in Figure 3, the parent molecule of all other retinoids [10].

Generally, they consist of a hydrophobic part, the central polyene linker, and a polar region (Figure 3). Synthetic derivatives were first created by modifying either the hydrophobic part and/or the polyene linker, as well as binding aromatic moieties to the polar heads to decrease drug sensibility to heat and light, causing oxidation. Many synthetic compounds were achieved by changing the β-ionone ring with aromatic ones, and/or cyclizing the polyene chain, thus reducing the conformational flexibility of the molecules. In this way the length and directionality of the molecule were better defined, and an energetic benefit when the molecule binds to a retinoid receptor was achieved. 

### 2.1. Retinoids Generations

Retinoids are commonly divided into three generations (Table 1), based on their molecular structure and receptor selectivity [11]. Some experts in the field acknowledge also a fourth generation of retinoids, which are derived from pyranones [3].

#### 2.1.1. First Generation Retinoids

**Figure 4:**
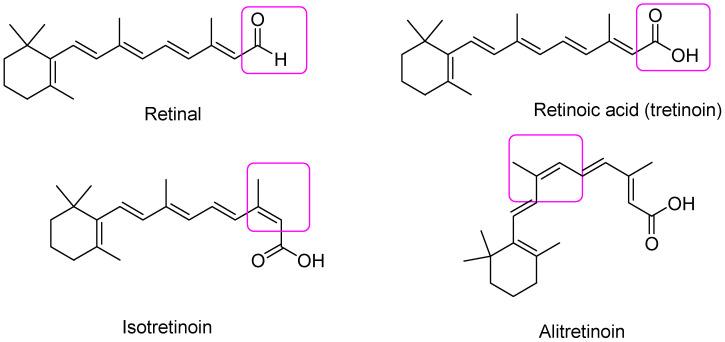
Chemical structure of first-generation retinoids. The modified groups respective to retinol have been highlighted in fuchsia squares.

Retinoids of the first generation include non-aromatic natural retinoids, such as retinol (vitamin A), shown in Figure 3, and its metabolites retinal, tretinoin (all-*trans*-retinoic acid (ATRA)), isotretinoin (13-*cis* retinoic acid), and alitretinoin (9-*cis* retinoic acid). Collectively, first-generation retinoids retain the cyclic structure of vitamin A with changes occurring in the polar group at the end of the side chain (Figure 4) [3]. 

The active metabolites of retinol, mainly all-trans-retinoic acid (ATRA), are important signaling molecules that are capable of inducing gene expression by activating specific nuclear hormone receptors [12], such as retinoic acid receptors (RARs) which form heterodimers with retinoid-X receptors (RXRs). It has been demonstrated that the metabolism of vitamin A is essential to preserve immune functions, promote good vision, and sustain the development, growth, and maintenance of several body tissues [13]. Incapable of synthesizing retinol, animals and humans are forced to obtain it from nutrients such as the carotenoids in fruits and vegetables, or by eating liver where carotenoids have already been processed and stored in the form of retinyl esters [13]. 

Chemical modification of the terminal-polar group of retinoids could be a useful way to reduce their toxicity, as well as a strategy to modify their activity, metabolism, and tissue distribution [14,15]. Among first-generation compounds, tretinoin (ATRA) was the first retinoid developed for clinical use as a topical agent. For example, tretinoin is water soluble up to concentrations of 210 nM at room temperature and pH 7.3 [12]. Tretinoin is effective in the treatment of acne vulgaris, photoaging, and rhytides. It is also used off-label to treat keratosis pilaris, actinic keratosis, and hyperpigmentation (melasma and solar lentigines) [16]. Tretinoin is present in different formulations, including creams and gels. Unfortunately, tretinoin is slightly irritating and is more photolabile than other retinoids [17]. In this regard, micro-formulations of tretinoin such as Retin-A Micro 0.04% and 0.1% have been developed using microsphere technology, thus improving photostability and mitigating some of its adverse effects [18]. Tretinoin has also been formulated as a combination product with the antibacterial clindamycin for the treatment of acne vulgaris.

#### 2.1.2. Second-Generation Retinoids 

**Figure 5:**
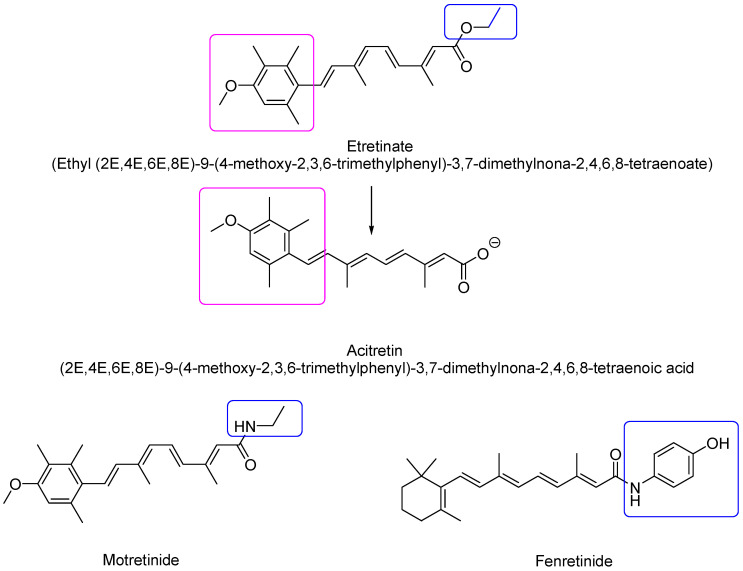
Chemical structure of second-generation retinoids. The modified groups with respect to retinol have been evidenced in fuchsia and blue squares.

Second-generation retinoids are synthetic monoaromatic compounds deriving mainly from changes to the cyclohexene ring of vitamin A. This generation comprises, among others, etretinate and acitretin (Figure 5) [19]. 

Due to the presence of the benzene ring instead of cyclohexene, these compounds are more hydrophilic than first-generation retinoids and consequently possess a higher bioavailability. Etretinate was developed as a medication approved by the FDA in 1986 by Hoffmann–La Roche (trade name Tegison). It is indicated for the treatment of severe psoriasis and other dyskeratoses [20]. It was subsequently removed from the Canadian market in 1996 and the United States market in 1998 due to the elevated risk of birth defects. It remains on the market in Japan as Tigason. Acitretin, the active orally bioavailable metabolite of etretinate, is derived from the hydrolysis of the ester function and has currently replaced etretinate in the treatment of various skin disorders. At physiological pH, acitretin is in the carboxylate form and thus contains a negatively charged side group which makes it less lipophilic than etretinate [3]. Its mean half-life elimination is two days, while the half-life of etretinate is 120 days. Additionally, some studies reported that etretinate can still be detected in the subcutis more than two years after discontinuation of therapy. Since acitretin can be in vivo converted to etretinate in the presence of ethanol, patients should abstain from drinking alcohol during therapy with acitretin and for at least a couple of months after stopping it. Motretinide is endowed by anti-keratinizing activity and is used as a topical agent in the treatment of acne [3]. N-(4-hydroxyphenyl)-retinamide or fenretinide (4-HPR) is a synthetic amide of ATRA first produced in the late 1960s, and will be discussed in depth in the following sections.

#### 2.1.3. Third-Generation Retinoids 

**Figure 6:**
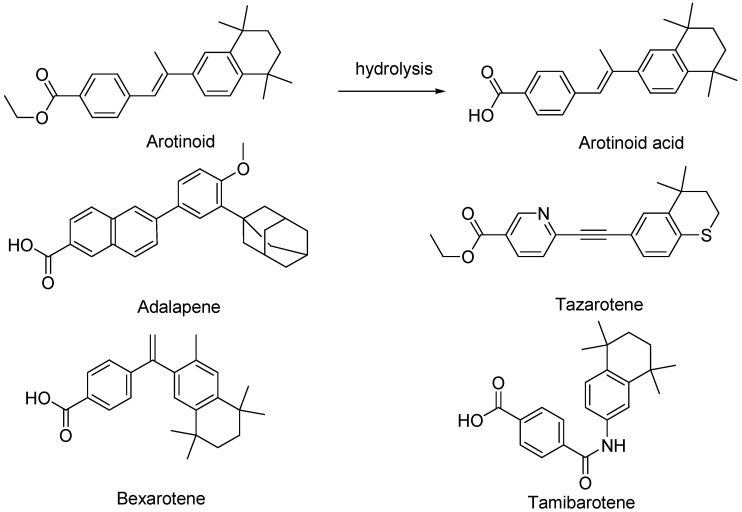
Chemical structure of third-generation retinoids.

Third-generation retinoids are polyaromatic molecules, obtained via cyclization reactions of the polyene side chain and include arotinoids (arotinoid ethyl ester and arotinoid acid), adapalene, tazarotene, bexarotene, and tamibarotene (Figure 6). 

Due to their more rigid structure with respect to first- and second-generation retinoids, third-generation retinoids bind a narrower variety of receptors [20]. Arotinoid acid is a non-clinically approved retinoid that consists of benzoic acid substituted at position 4 by a 2-(5,5,8,8-tetramethyl-5,6,7,8-tetrahydronaphthalen-2-yl)-prop-1-en-1-yl group. It acts as a selective agonist for the retinoic acid receptors (RAR). It has a role as an antineoplastic agent, a retinoic acid receptor agonist, and a teratogenic agent. Not yet approved as therapeutic, arotinoid ethyl ester (RO 13-6298) is a very potent retinoid that exerts a profound influence on epithelial and mesenchymal differentiation in doses 500 times lower than those of compounds of the first and second retinoid generation [21]. Tazarotene has been formulated as a cream and gel (0.05% and 0.1%) for topical administration. Currently, it is the most potent available retinoid indicated in the treatment of acne vulgaris and plaque psoriasis [19,22]. In the latter case, tazarotene is marketed as a lotion in combination with halobetasol, a high-potency topical corticosteroid. Adapalene is formulated for dermal administration to treat acne vulgaris, also in combination with benzoyl peroxide. It is also used off-label in the treatment of hyperpigmentation, actinic keratosis, photoaging, and rhytides [16]. The cream and gel are available in 0.1% and 0.3% concentrations, respectively, while gels are also available over the counter (OTC) in the United States. Adapalene is the least irritating and least disposed to photodegradation retinoid, allowing for daytime application [17]. Bexarotene (Targretin) is a retinoid functioning as an antineoplastic agent indicated by the FDA for Cutaneous T cell lymphoma [23]. It was approved by the U.S. Food and Drug Administration (FDA) in December 1999, and the European Medicines Agency (EMA) in March 2001. It is available as a generic medicine. It has been used off-label for lung cancer, breast cancer, and Kaposi’s sarcoma. Bexarotene acts selectively by activating retinoid X receptors (RXRs), as opposed to the retinoic acid receptors (RAR), the other major target of retinoic acid [24,25]. Chemically, tamibarotene is a dicarboxylic acid monoamide resulting from the condensation of one of the carboxyl groups of terephthalic acid with the amino group of 5,5,8,8-tetramethyl-5,6,7,8-tetrahydronaphthalen-2-amine. Tamibarotene is an orally active novel synthetic retinoid acting as an antineoplastic agent and a retinoic acid receptor α,β agonist, which was developed to overcome ATRA resistance [26]. It has been demonstrated that tamibarotene is approximately ten times more potent than ATRA in inducing cell differentiation and apoptosis in HL-60 (human promyelocytic leukemia) cell lines in vitro, showing sustained plasma levels compared to ATRA, and exhibiting a lower toxicity profile. Currently approved in Japan for the treatment of recurrent APL, tamibarotene is undergoing clinical trials in the United States [26].

#### 2.1.4. Fourth-Generation Retinoids

**Figure 7:**
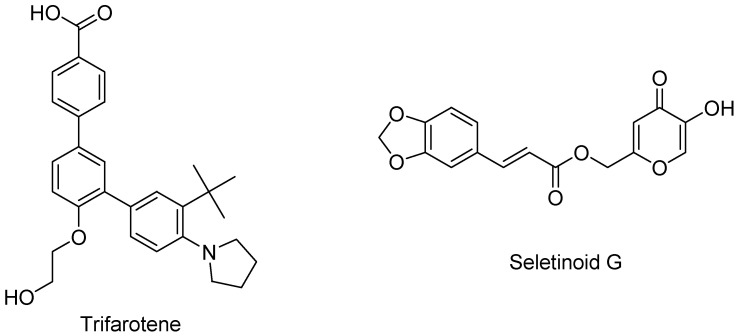
Chemical structure of fourth-generation retinoids.

Trifarotene (Figure 7) is a fourth-generation retinoid selective towards the RAR γ receptor located in the epidermis, marketed for topical administration [27]. 

Trifarotene is formulated as a cream suitable for treating acne vulgaris of the face and trunk. Studies testing the possible systemic absorption of trifarotene demonstrated unquantifiable levels within the target populations ≥18 years and pediatric patients (9–17 years) with moderate to severe acne. Seletinoid G is a novel pyranone derivative having the chemical name 2-((3E)-4(2H,3H-benzo[3,4-d]1,3-dioxolan-5-yl)-2-oxo-but-3-enyloxy)-5-hydroxy-4H-pyran-4-one. It was developed in the year 2005 by Kim et al. as a novel synthetic retinoid [28]. Although still not clinically approved, seletinoid G could be a potent anti-aging agent for protecting the skin barrier [29].

## 3. Fenretinide (4-HPR)

Although some authors insert *N*-(4-hydroxyphenyl)-retinamide (4-HPR), or fenretinide (FEN), in the group of third-generation retinoids [30,31], being a monoaromatic compound maintaining the linear polyene chain, it is more correct to insert 4-HPR in the second-generation group of retinoids. 4-HPR is a derivative of ATRA forming via amidation of its carboxylic group with 4-hydroxyphenyl aniline (Figure 5). The additional 4-hydroxyphenyl group is thought to be responsible for several beneficial effects deriving from treatment with 4-HPR, with respect to the use of ATRA. While ATRA and retinyl-acetate, when supplemented in large doses, showed liver toxicity associated with prolonged exposure, thus limiting their potential use as medicinal agents, 4-HPR has demonstrated a decreased toxicological profile [13]. Indeed, chronic treatment with retinyl-acetate causes deposition of retinyl esters in the liver with consequent hepatic toxicity, while 4-HPR does not accumulate in the rats’ liver [32]. On the contrary, 4-HPR and its metabolites are preferentially stored in fatty tissues such as the mammary gland [32,33], thus preventing the emergence of hepatotoxic accumulation. The specific accumulation of 4-HPR in fatty tissues could be beneficial for the prevention/treatment of breast cancer, obesity, and type II diabetes [32,33,34,35]. Studies carried out on rats and rabbits have revealed that when orally administered at a dose of 20 mg/kg/d, 4-HPR did not provide adverse effects in both species. Higher doses of 125–800 mg/kg/d were only weakly teratogenic in these species [36]. Also, experiments carried out using hamsters administered with up to 130 mg/kg of the 4-HPR isomer 13-*cis*-*N*-(4-hydroxyphenyl)retinamide failed to induce a teratogenic response [37]. Collectively, since it is unable to induce point mutations or chromosomal aberrations, 4-HPR can be considered a non-genotoxic compound, with potential therapeutic applications higher than those of ATRA.

### 3.1. Pleiotropic Effects Triggered by Fenretinide (4-HPR)

**Figure 8:**
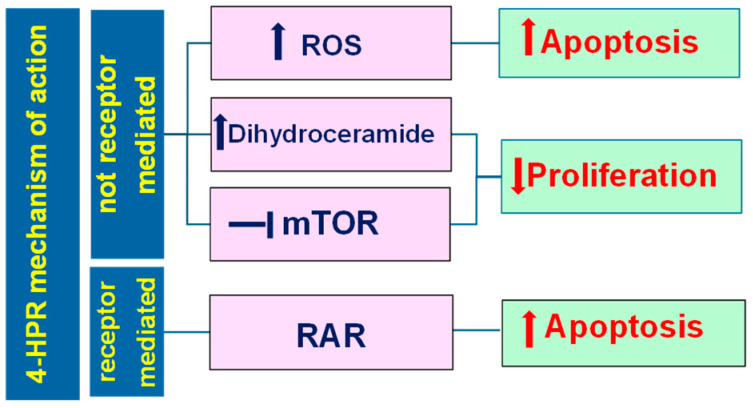
Main and recognized mechanisms of action of 4-HPR responsible for cancer cell inhibition. Pointed arrows represent pathway activation, and blunt arrows represent pathway inhibition.

Although not completely understood, 4-HPR mechanisms of action can be divided into two macro-classes: receptor-dependent and receptor-independent. Typically, retinoids exert their effects by the activation of nuclear retinoic acid receptors (RARs) and retinoic X receptors (RXRs), in turn classified as α, β, and γ. These receptors are ligand-activated transcription factors regulating several cellular processes, including growth, differentiation, and apoptosis, and make up part of the steroid hormone receptor superfamily [38]. In addition, 4-HPR can exert antitumor effects by a more complex combination of different processes, including at least four different mechanisms of action (Figure 8) [39].

Collectively, unlike ATRA, 4-HPR induces its anticancer effects mainly via retinoic receptor-independent mechanisms, and the anticancer effects of 4-HPR deriving from its binding to RARs represent only 15% of those observed with ATRA treatment. In fact, although 4-HPR can also induce RAR transcriptional activation, it binds very poorly to all three RAR isoforms [40], probably due to the absence of the carboxyl function in its structure, and is thus more than 100-fold less potent than ATRA by this route [41]. Particularly, the binding of 4-HPR to the RAR β receptors increases their expression and induces the translocation of the nuclear receptor Nur77 from the nucleus to the cytoplasm. Here, Nur77 binds the anti-apoptotic Bcl-2 genes, allowing their conformational change towards pro-apoptotic structures that expose the BH3 domain [42,43,44,45].

Additionally, the generation of ceramides has garnered considerable attention as another possible mechanism by which 4-HPR induces the death of cancer cells [46]. In particular, 4-HPR can increase the levels of potentially cytotoxic dihydroceramides (DhCers) in a dose- and time-dependent manner, through both stimulation of de novo synthesis and inhibition of dihydroceramide desaturase 1 (DES1). DES1 inhibition leads to an increase in the DhCer/Cer ratio in cell membranes that causes stress to the endoplasmic reticulum (ER) and blocks the phosphatidylinositol-3-kinase (PI3K)/Akt. Consequently, (PI3K)/Akt becomes unable to activate the mammalian target of rapamycin (mTORC1), thus inhibiting the NF-kB signaling pathways.

Additionally, the ER stress induces phosphorylation of the eukaryotic initiation factor 2 (eIF2) by PERK, thus inhibiting its activity as an initiator of protein translation [47]. All these processes lead to the inhibition of cell proliferation. Another mechanism of action of 4-HPR leading to proliferation inhibition starts with the inhibition of the mammalian target of rapamycin (mTORC2), as confirmed by the evidence that the knockdown of this protein kinase in cancer cells decreases the cytotoxicity of 4-HPR [48]. 4-HPR is believed to directly bind the ATP-binding niche of both the mTORC1 and mTORC2 proteins through hydrogen bonds and hydrophobic interactions, thus suppressing both directly and indirectly the activities of both the mTORC1 and the mTORC2 complexes and the associated PI3K/AKT pathway, with a consequent proliferation decrease [39].

The fourth mechanism depends on the capability of 4-HPR to replace retinol in the mitochondrial signalosome, made up of a signal adaptor protein (p66Shc), protein kinase Cδ (PKCδ), and cytochrome C (CytC). The retinol/p66Shc-PKCδ-CytC signalosome catalyzes the transfer of electrons from PKCδ to CytC and vice versa [39]. When the electron is donated by PKCδ to cytochrome, the zinc-finger domain of the kinase is oxidized, with the activation of pyruvate dehydrogenase kinase 2 (PDK2) through phosphorylation, thus allowing pyruvate dehydrogenase phosphatase 1 (PDP1) and 2 (PDP2) to activate the pyruvate dehydrogenase complex (PDHC) that feeds the tricarboxylic acid cycle (TCA) [39]. This pathway renders NADH available for oxidative phosphorylation (OXPHOS) reactions with the generation of ROS and ATP.

In the presence of physiological concentrations of retinol, this process is reversible, and ROS production is controlled. By contrast, when retinol is replaced by 4-HPR or ATRA, the mechanism becomes irreversible and the PDHC is locked in an activation state, leading to uncontrolled ROS overproduction [39].

The aberrant ROS increase leads to mitochondrial membrane permeabilization and activates the p38 apoptotic pathway with CytC release, caspase-9 activation with induction of apoptosis, and cell death [49].

In cancer cells, while apoptosis is activated in the presence of high ROS levels, low levels activate the JNK autophagy pathway, being the cellular response governed by a redox sensor protein that detects ROS concentration (DJ-1) [39].

Collectively, high concentrations of 4-HPR should promote apoptosis by generating high ROS levels, while low concentrations of the drug should favor cell survival over cell death. This relationship between concentration and type of response has been observed in glioma cell lines treated with 4-HPR. In particular, 4-HPR induced apoptosis at concentrations of 10 μM and autophagy at 5 μM, while at concentrations higher than 10 μM, the glioma cells both underwent apoptosis and showed the characteristic features of autophagy [50].

In addition, 4-HPR exerts antiangiogenic activity through both a direct effect on endothelial cell proliferative activity and an inhibitory effect on the responsivity of the endothelial cells to the proliferative stimuli mediated by angiogenic growth factors [51].

Taken together, these findings suggest that 4-HPR can activate a multifactorial program in cancer cells composed of signals of autophagy, apoptosis, and proliferation inhibition. Given these considerations, the enhancement of drug bioavailability appears of paramount importance to obtain significant responses in in vivo treatments.

### 3.2. Results of Capsular 4-HPR Formulation in Early Clinical Trials

As introduced above, 4-HPR was first synthesized with the aim of obtaining a retinoic acid (ATRA) derivative with less adverse effects to be applied in dermatology. Unexpectedly, 4-HPR was found inactive in dermatologic applications, therefore interest in this molecule waned until the discovery of the antitumor activity of ATRA (tretinoin). Indeed, the use of retinoids in cancer changed significantly when Ted Breitman at the National Institutes of Health demonstrated that ATRA could convert leukemia cells into normal neutrophils by a differentiative effect [52]. These findings paved the way for the use of retinoids in cancer treatment and ended in 1995 with the approval of ATRA for the treatment of acute promyelocytic leukemia (APL), a previously fatal disease, by the FDA (Food and Drug Administration) and the European Medicines Agency (EMA), as indicated in Table 2 [53].

Among endogenous retinoids, 13-*cis* retinoic acid (isotretinoin), which can be mutually interconverted to ATRA by isomerase in the human body, is currently incorporated in the standard therapeutic regimen as a maintenance therapy for high-risk neuroblastoma (HRNB) following the publication of the positive results of the CCG-3891 trial [58]. No formulation with such indications has been marketed so far, but oral-only capsules for the treatment of severe acne were approved by the FDA and EMA in 1982 and 2003, respectively [54]. Among synthetic retinoids, bexarotene was approved by the FDA (1999) and the EMA (2001) for use against refractory cases of cutaneous T-cell lymphoma [55], while 4-HPR [56] and tamibarotene [57] are currently being evaluated for the treatment of neuroblastoma.

Concerning 4-HPR, the long list of clinical trials aimed at proving its efficacy and tolerability started early in 1990 with a phase II study involving 31 patients with advanced breast cancer and melanoma. Only minimal activity of 4-HPR was observed in this study, since two patients achieved mixed responses (6.7%), and eight (26.7%) had disease stabilization. Neither a partial nor a complete response was attained at a drug oral dose of 300–400 mg/d [59].

In another phase II trial study carried out to evaluate 4-HPR efficacy in metastatic renal carcinoma, the capsular formulation was administered at a dose of 900 mg/m^2^ twice a day for 7 days in a 21-day cycle for 76 cycles. No complete response was noted and 4-HPR showed minimal activity overall, consistent with the low drug levels detectable in the tumor tissue [60]. In only three patients, the drug reached the concentration of 3.6 µM (1.4 µg/g), 3.8 µM (1.5 µg/g), or 7.9 µM (3.1 µg/g), while in the remaining cases, 4-HPR levels were below the limit of quantification.

A clinical trial to assess the systemic toxicity of oral 4-HPR in pediatric patients, treated with high-dose schedules, evidenced that the maximum dose tolerated (MTD) was 2450 mg/m^2^/d (divided thrice daily, for 7 days, every 21 days) [61]. The study reported that 10 μM 4-HPR plasma levels were achieved without significant toxicity. This limited toxicity paved the way for another phase I clinical study in which the dose was increased up to 4000 mg/m^2^/d (single-day dosing, for 4 weeks, every 5 weeks) [8].

In this study, Garaventa et al. reported that after the first dose administration, the average 4-HPR peak plasma levels reached about 6 μM and increased by 2-fold after a 28-day treatment [8]. Commonly observed toxicities included skin xerosis and nyctalopia. Only one patient experienced elevated levels of transaminases, while three patients developed diarrhea. All these adverse reactions were rapidly resolved by discontinuing the treatment. Overall, the clinical trial confirmed the high tolerability of the drug but ended without determining the MTD due to the patient’s poor compliance in assuming an extremely high number of capsules. Additionally, it is worth pointing out that the oral formulation consisted of 100 mg 4-HPR in corn oil (704 mg) and polysorbate 80 (60 mg), although polysorbates may induce kidney and liver failure with an MTD of 10 mg/kg bw in young children [62].

Only one Phase II study has been reported by Villablanca et al., in which 4-HPR was used for the treatment of neuroblastoma [63]. Oral capsular 4-HPR was administered to 62 patients for 7 days every 21 days (2475 mg/m^2^/d or 1800 mg/m^2^/d) for a maximum of 30 cycles of treatment. Mean 4-HPR steady-state trough plasma concentrations were 7.25 μM at day 7 of course 1. As expected, toxicities were mild and reversible. As the authors of this study concluded, responses were difficult to interpret due to the low micromolar drug plasma concentrations reached (6–13 µM) and the high interpatient variability. As one of the main limitations to the clinical development of orally administered 4-HPR was the achievement of effective and consistent plasma concentrations in patients, the study of the metabolism of the parent drug clearly appeared to be an important issue. Cytochrome P450 enzymes including 2C8, 3A7, 3A5, 2C18, 3A4, and 2C9 are involved in ATRA metabolism, while 2C8, 3A7, 4A11, 1B1, 2B6, and 2C9 are responsible for the metabolism of 13-cisRA [64]. 4-HPR metabolism is similar to that of ATRA and 13-cis retinoic acid and produces the active metabolite, 4-oxo fenretinide (4-oxo 4-HPR), and the inactive metabolite 4-methoxy fenretinide (4-MPR) responsible for the reduced responses observed in the clinical trials [64].

Otherwise, 4-HPR was successfully used to prevent pre-menopausal breast cancer in approximately three thousand high-risk Italian women in a 5-year clinical trial. 4-HPR was tested at an oral daily dose ranging from 200 to 900 mg, resulting in 1–3 μM drug plasma levels with minimal toxicity [65]. It was observed that 4-HPR induces a significant risk reduction in second breast cancer in premenopausal women, which is remarkable at younger ages, and persists several years after treatment cessation. Particularly, the younger the women, the greater the risk reduction. However, these results were never pursued or studied further in any significant way, as 4-HPR failed to displace tamoxifen in this setting and interest waned. Additional pediatric and adult clinical trials in which 4-HPR has been tested for cancer treatment are reported in Table 3, some of which are discussed in detail in the following sections.

### 3.3. Subsequent Attempts to Raise Drug Bioavailability

To date, the problem of how to deliver a safe and effective dose of 4-HPR to achieve adequate therapeutic blood levels in a cancer patient has remained unsolved. Over the years, intensive basic research studies aiming both at investigating the specific mechanisms of action of 4-HPR and realizing new formulations have been reported [74]. Many new aspects of the cellular pathways triggered by 4-HPR have been revealed and described, most notably those leading to cell apoptosis [75]. Such studies have stimulated many new attempts to find a more effective, bioavailable dosage form of 4-HPR for cancer treatment.

#### 3.3.1. Oral Formulations

The first attempt was performed by Maurer et al. and focused on the preparation of a waxy powder to be either directly orally administered or mixed with food, to deliver it more easily to younger patients [76]. The formulation was prepared by adding the drug to a lipid matrix named LYM-X-SORB (lysophosphatidylcholine, monoglycerides, and free fatty acids, 1:4:2), at a 4-HPR/LYM-X-SORB molar ratio of 0.8:1.0, able to form chylomicron-like micelles in the stomach. To ameliorate the palatability, the powder was blended with sugar and wheat flour. The 4-HPR LYM-X-SORB formulation was able to increase the drug plasma levels by 4 times with respect to capsules and significantly prolonged the drug survival in human neuroblastoma murine xenografts [76]. These preclinical studies paved the way for a phase I trial on relapsed/refractory neuroblastoma patients to determine the MTD and the pharmacokinetics of 4-HPR delivered from the lipid matrix [56]. The oral powder was given at a dose expected to achieve plasma levels like those of the capsular phase II dose of 352 mg/m^2^/d. This dose was then incremented up to 2210 mg/m^2^/d, divided into two doses daily, for 7 consecutive days, every three weeks. The trial demonstrated that 4-HPR LYM-X-SORB powder provided from two- to six-fold higher drug plasma levels (at least 15 µM) than equivalent doses delivered using corn oil capsules. In particular, the day 6 mean peak 4-HPR plasma level at 1700 mg/m^2^/day was 21 µM. The study reported minimal toxicity, while the MTD was not reached, so the authors recommended a phase II schedule of 4-HPR LYM-X-SORB powder of 1500 mg/m^2^/d, divided into three doses, on days 0–6, of a 21-day course. Since inter-patient variability was still observed, reflecting the presence of high and low metabolizers, the necessity of a concurrent administration of a P450 inhibitor (i.e., ketoconazole) was established. Complete tumor responses were observed only in patients with tumor involvement limited to bone marrow and/or bone metastases. The same formulation was also concomitantly tested in adults with refractory solid tumors [77]. The study revealed that the formulation was less tolerated, and a dose de-escalation was needed. The MTD was established at 800 mg/m^2^/d, due to patients’ particular sensitiveness to the LXS matrix. Variable 4-HPR plasma levels were achieved, and concomitantly comparable 4-MPR concentrations emerged along with drug repeated administrations. Moreover, the drug absorption and mean plasma levels, probably limited by gastrointestinal (GIT) irritation, were generally no better than those achieved with higher doses (1800 mg/m^2^/d) of capsular 4-HPR.

Orienti et al. prepared a nanoencapsulated 4-HPR formulation (HPR-NC) containing glucosamine butyrate as a gastrointestinal availability enhancer, expected to promote both drug solubilization and absorption, and gelatin as a matrix forming agent [78]. Glucosamine was conjugated with butyric acid because low molecular weight fatty acids and sugar moieties are both known to interact with the intestinal epithelium, enhancing its permeability. Moreover, the resulting amphiphilic structure is expected to improve 4-HPR solubilization in the aqueous environment of the gastrointestinal tract (GIT) [78]. The loaded nanocapsules were obtained by mixing a 4-HPR solution in ethanol, containing glucosamine butyrate, with an aqueous solution of gelatin. Coprecipitation of the drug and gelatin at the interface of the micelles formed by the emulsifier, triggered by the ethanol extraction towards the aqueous phase, led to the formation of nanocapsules [78]. In vivo absorption studies and in vivo efficacy were tested in neuroblastoma-bearing mice in feeding and fasting conditions. Mice were gavaged with HPR-NC at doses ranging from 25 to 200 mg/kg, 3 days/week for a total of 4 weeks. At the highest dose, HPR-NC provided a significant reduction in tumor growth with respect to the capsular 4-HPR at the same dose. Moreover, fasting conditions strongly improved the antitumor activity.

In a patent by Laurent Pharmaceuticals, tablets made of a spray-dried amorphous solid dispersion of polyvinylpyrrolidone (PVP)-4-HPR were reported [79]. In a pharmacokinetic study in rats exposed to 20 mg/kg 4-HPR, the formulation exhibited improved bioavailability, and more specifically an AUC_0–48_ 4 times higher than that of capsular formulation (5911 ng × h/mL vs. 1490 ng × h/mL, respectively), as well as a C_max_ value of 505 ng/mL vs. 125 ng/mL of the corn oil formulation [79]. 

Another attempt to provide a more bioavailable drug formulation involved the complexation of 4-HPR with 2-hydroxypropyl β-cyclodextrin after its deprotonation [80]. The formation of the inclusion complex raised total drug solubility up to 2410 µg/mL, thus increasing drug concentration in solution by 1409-fold. To increase the aqueous solubility of 4-HPR, its potassium salt was prepared and dissolved in the presence of the complexing agent. The formulation, namely NanoFEN, was patented and its bioavailability after oral administration was assessed and compared to capsular 4-HPR. In the pharmacokinetic study, animals were gavaged with a dose of 5 mg/kg. The C_max_ value was more than 2-fold higher than that provided by the capsular formulation (730 ng/mL vs. 298 ng/mL) and AUC_0–last_ was 3-fold greater (9378 h × mg/mL vs. 3201 h × mg/mL). In vitro studies on LCSC6 revealed widespread inhibition of the mTOR pathway, in parallel with a massive accumulation of bioactive dihydroceramide lipids, indicating that NanoFEN was able to activate the multifactorial program in cancer cells. In vivo studies aimed at investigating the therapeutic efficacy of NanoFEN were performed in colon and lung cancer xenografts, following intraperitoneally drug injection and compared with other antitumor drugs. Xenografts treated with chemotherapeutics showed an accelerated growth of tumor mass, while NanoFEN was significantly more efficacious in decreasing tumor proliferation. However, as the antitumor activity was not essayed after oral administration, these results are more comparable to a parenteral administration than to an oral one.

The same authors prepared nano-micelles by ion pair formation between 4-HPR and phosphatidylcholine as counter ion [81]. The resulting formulation, Bio-nFeR, was able to increase 4-HPR water concentration by a linear trend, typical of the solubilization mechanism provided by amphiphilic excipients (glyceryl tributyrate and phosphatidylcholine). The maximum concentration of Bio-nFer evaluated (300 mg/mL) led to 25.41 mg/mL of 4-HPR in solution. A pharmacokinetic study showed that a single oral administration at 200 mg/kg provided a C_max_ of 9.2 µM. In vivo studies confirmed the superior antitumor activity of the micellar formulation with respect to the capsular 4-HPR in subcutaneous lung, melanoma, and colon tumor models [81].

All the oral formulations developed so far and evaluated in vivo are summarized in Table 4. The capsular formulation was taken as a reference and was also inserted in the table, to allow an easier comparison.

#### 3.3.2. Parenteral Formulations

The main parenteral formulations developed so far and evaluated in vivo are reported in Table 5.

One of the first intravenous formulations of 4-HPR was obtained by its conjugation with polyvinyl alcohol (PVA, Mw = 10,000 Da) via a carbonate bond [82]. The most substituted PVA provided a 200-fold increase in the total drug solubility of 343 µg/mL. The synthesized amphiphilic structures were able to self-assemble in water, generating aggregates of 250–500 nm and providing drug release rates not exceeding 20% in the first 6 h and almost constant in the subsequent period. The in vivo antitumor activity was tested in a neuroblastoma metastatic tumor model at a 4-HPR dose of 13.5 mg/mL administered five times every 3 days [82]. Notably, the equivalent dose of free 4-HPR could not be injected because of the high content of ethanol needed for drug solubilization. The in vivo results showed a significant increase in the mean survival time vs. control mice, suggesting the potential ability of this conjugate to treat the residual minimal disease responsible for the relapsing of neuroblastoma [82].

A poly-(ethylene glycol)–poly(aspartate) block copolymer modified with benzyl groups was employed to encapsulate 4-HPR into polymeric micelles [83]. These micelles possessed a hydrophobic inner core able to entrap lipophilic drugs and a hydrophilic shell determining their circulation time in the hematic flow and thus their ability to accumulate in tumor tissue. The micelles were prepared via solvent casting method by dissolution of the drug and the copolymer in chloroform. After hydration, the drug delivery system showed an EE% of 70% and a mean particle size of 173 nm. The in vivo distribution study performed in mice by injecting intravenously a dose of 75 mg/kg showed a maximum tumor concentration of 4-HPR of 56.6 μg/g after 6 h and an enhancement of drug retention in plasma that could significantly inhibit tumor growth in a subcutaneous melanoma model.

A liposomal 4-HPR system engineered with an active targeting moiety directed to tumor endothelial cells was intravenously injected in orthotopically xenografted neuroblastoma-bearing mice at a dose of 1 mg/kg twice a week for 6 weeks [84]. Liposomes, prepared by the reverse phase method, were made of hydrogenated soy phosphatidylcholine (HSPC), cholesterol (CHE), 1,2-distearoyl-glycero-3-phosphoethanolamine-N-[methoxy(polyethyleneglycol)-2000] (DSPE-PEG2000) and grafted with NGR peptide, able to recognize the specific isoform of aminopeptidase N (APN) (CD13)-positive tumor vasculature. The formulation showed a good encapsulation efficiency (EE%) and negligible drug leakage in 25% plasma at 37 °C during the first 72 h [84]. In vivo experiments clearly evidenced the enhancement of the therapeutic efficacy provided by the targeted liposomes [84]. These results confirmed that associating the drug’s antitumor effect with vascular targeting may be a valid strategy to increase drug accumulation in the tumor tissue, otherwise limited by the high hydrostatic pressure produced by the enhancement permeability and retention (EPR) effects [84].

Human Serum Albumin (HAS) was used as a complexing agent for 4-HPR formulation with the aim of improving its bioavailability by increasing its water solubility. Additionally, the affinity of HAS to Caveolin-1, whose overexpression is correlated with a poor prognosis in non-small cell carcinomas, was strategically exploited [85,86]. The rationale for using HSA was based on its long half-life in the body associated with the fact that tumors are able to trap plasma proteins and utilize their degradation products as a source of energy for proliferation [86]. Moreover, HAS is endowed with high stability and is easy to handle, characteristics that make it a very promising drug delivery system. The complex was prepared by adding a 4-HPR ethanolic solution to a HAS aqueous solution. By mixing the two solutions, a suspension formed, which was subsequently subjected to sonication and filtration to reduce particle size. Finally, the solution containing 4-HPR and HAS was lyophilized and nanocapsules of 80–100 nm were obtained [85]. In vitro studies aimed at elucidating the carrier uptake from A549 cells suggested the presence of an endocytosis mechanism based on caveolar vesicles. Upon injection by the tail vein at the dose of 1 mg/kg, 12 times every three days in a xenograft model of A549, the subcutaneous tumor masses underwent a significant reduction in the tumor volume of 63% with respect to control, according with the high drug concentration detected in the tumor tissue (5.7 µM) at the end of the experiment. After 20 days, tumor growth was significantly slower in the treated mice than in the control group and this trend persisted until the mice were euthanized [85].

To provide a sustained release of 4-HPR, microspheres were prepared using copolymers of lactic and glycolic acids (PLGA), in the form of an injectable drug delivery system [87]. The drug delivery system was prepared by the standard oil-water emulsion with the use of methylene chloride as an oil phase containing PLGA and emulsifier, and a water solution containing PVA 0.5% as an external phase. However, as 4-HPR is insoluble in the selected organic solvent, the oil phase consisted of a drug dispersion. The surfactants were added to enhance 4-HPR solubility and release. SEM pictures highlighted the presence of drug crystals on the surface of the microspheres without surfactants. In contrast, when emulsifiers were used, they concentrated close to the interface O/W, incorporating the outer drug molecules. To further raise the release rate, a pore-forming salt (MgCO_3_) was employed and added to the oil phase. The drug delivery profiles were characterized by a massive burst release ranging from 36% to 50%, followed by a slow sustained release up to 28 days. To assess the in vivo drug release, two formulations, one containing 20% Brij 98 and one containing 3% MgCO_3_, were administered to animals in a 0.2 mL intramuscular injection of the microsphere suspension containing 4.2 mg 4-HPR. The best results were obtained with the formulation incorporating 3% MgCO_3_, and 20% drug loading, where the C _max_ reached the value of 0.24 µM on the 10th day. However, the drug plasma level was lower than the free 4-HPR suspension, but the T _max_ for the microsphere formulations was much longer compared to the drug suspension (240 h vs. 24 h), corroborating the different release mechanism (polymer degradation vs. drug dissolution). The relative bioavailability calculated from the area under the curve (AUC) was 101%, indicating a complete drug release after 4 weeks. However, the drug release was very slow, as expected for this type of delivery system. Therefore, in our opinion, the performance of these microspheres may not fit with the requirement of a high amount of free drug available at the tumor site.

More recently, a phase I study was conducted to determine the MTD, dose-limiting toxicities (DLT), and pharmacokinetics of a 4-HPR-loaded O/W intravenous emulsion in patients with relapsed/refractory hematologic malignancies [73]. 4-HPR was administered by continuous infusion at doses ranging from 80 to 1810 mg/m^2^/d, on days 1 to 5 in 21-day cycles. The MTD was reached at 1280 mg/m^2^/d for 5 days. Steady-state plasma levels were approximately 10 µg/mL at 640 mg/m^2^/d, 14 µg/mL at 905 mg/m^2^/d, and approximately 22 µg/mL at 1280 mg/m^2^/d [73]. The pharmacokinetic analysis showed a mean steady-state drug plasma level in the 50 µM range, thus demonstrating that the intravenous delivery method may circumvent the problem of the low plasma 4-HPR concentrations achieved after oral administration. Promising sustained complete and partial responses were observed in heavily pretreated patients with T-cell lymphomas. However, this preparation was reported to contain significant amounts of egg phospholipids and soybean oil which caused dose-limiting toxicities due to hypertriglyceridemia. Moreover, the concentration (or potency) of 4-HPR in this drug formulation was relatively low, and drug infusion must be administered over a relatively prolonged period of time (1 day at an in-patient clinic). Subsequently, the same formulation was tested for its efficacy against solid tumors in 23 patients [88]. The initial starting dose was chosen at 1280 mg/m^2^/d for 5 consecutive days in a 21-day cycle, based on the previous phase I trial in hematologic malignancies. However, the initial starting dose was decreased to 905 mg/m^2^/d, or to 724 mg/m^2^/d in case of significative adverse events (hypertriglyceridemia, fatigue, aspartate aminotransferase (AST)/alanine aminotransferase (ALT) increase, thrombocytopenia, bilirubin increase, and dry skin). Plasma samples revealed drug levels of 9.9 µg/mL at 905 mg/m^2^/d and approximately 10.8 µg/mL at 1131 mg/m^2^/d. A total of 28% of patients had stable disease as best response, 72% of patients had progressive disease, and no patients had objective responses, suggesting that the 4-HPR single-agent activity was minimal and combinatorial approaches were needed.

A much safer parenteral nano-formulation based on cyclic oligosaccharides was subsequently developed. The formulation, namely NanoFEN, exploited the ability of β-cyclodextrin to host lipophilic molecules like 4-HPR. NanoFEN was concomitantly assayed for its antitumor activity after both oral (as seen above) and intravenous administration [80]. The pharmacokinetics studies were performed in mice that received 5 mg/kg of 4-HPR intravenously. The C_max_ value was 6932 ng/mL for NanoFEN vs. 4134 ng/mL for free 4-HPR, and 298 ng/mL for capsular 4-HPR. The in vivo antitumor effects were tested in xenografts of lung and colorectal cancer at the dose of 10 mg/kg/three weekly/intraperitoneally and compared with those of other chemotherapeutics like cisplatin plus gemcitabine or oxaliplatin plus 5-fluorouracil. The tumor masses were significantly reduced when treated with NanoFEN compared to free 4-HPR, and the differences were markedly more evident after treatment cessation. Moreover, a long-term treatment of 70 days succeeded in establishing the ability of this formulation to prevent tumor recurrence [80].

A new nano micellar complex was prepared by combining 4-HPR with a quaternary amphiphilic amine (C16-ceramide) also endowed with antitumor activity [89]. RC16+ contains a polycyclic delocalized quaternary ammonium and a 16-carbon alkyl tail. The delocalization of the charge should reduce the binding to serum proteins and allow drug accumulation in the tumor tissue by the EPR effect. The complex (Fen-RC16+) strongly increased the total aqueous solubility of 4-HPR up to 1500 µg /mL and provided a cytotoxic effect on neuroblastoma cell lines resulting from the intrinsic activity of both the complex components. Moreover, the mean size of the nano micellar complex ranged from 20 nm to 40 nm, while the positive superficial charge induced adsorption of the complex on the tumor cell surface improving the intracellular uptake of 4-HPR. The in vivo antitumor effects of the cationic formulation were evaluated on SH-SY5Y xenografts. The Fen-RC16+ was administered at the dose of 2 mg/kg corresponding to 1.02 mg/kg 4-HPR and 0.98 mg/kg RC16+ every other day for a total of 8 administrations, evidencing a significant inhibition of tumor growth even after the treatment withdrawal.

#### 3.3.3. Transdermal Formulations

When possible, local drug delivery may represent the first choice of treatment since the therapeutic potential of a drug can be easily improved by direct administration to the pathological site. This is the case with the treatment of head and neck squamous cell carcinoma (HNSCC) dysplastic lesions. To this end, a mucoadhesive patch consisting of an adhesive layer (hydroxypropyl methylcellulose/polycarbophil in weight ratio 3/1), and drug release layers (5 wt% 4-HPR/Eudragit^®^ RL-PO/40 wt% sodium deoxycholate/20 wt% Tween^®^ 80), was assembled onto a backing layer and investigated for co-incorporation of tissue enhancers like propylene glycol and menthol [90]. A mucoadhesive patch co-incorporated with 2.5 wt% propylene glycol and 5 wt% menthol was found to be the optimal formulation for the oral mucosal drug permeation enhancement without affecting local irritation. The levels of 4-HPR delivered to rabbit buccal mucosa from a loaded patch ranged from 7.75 μg/g (19.8 μM) after 0.5 h to 108.2 μM (42.36 μg/g) after 6 h. Therefore, the patch application was able to provide therapeutically relevant concentrations in the targeted oral epithelium in a short treatment time, thus facilitating patient compliance.

#### 3.3.4. Subcutaneous Formulations

Long-acting release delivery systems may be a useful tool to prevent the need for high daily doses, especially in cancer chemoprevention or to contrast tumor recurrence. In this regard, 4-HPR solubility was enhanced by developing an amorphous solid dispersion (ASD) of the drug into polyvinylpyrrolidone (PVP) coated with PLGA [91]. An optimized formulation was prepared with PVP/drug 9/1 (*w*/*w*), and triethyl-acetyl-citrate (TEAC) as a plasticizer. Milli-cylindrical implants were designed to specifically provide a delayed release for circumventing the low patient compliance associated with parenteral administrations. ASDs were able to provide super-saturated drug solutions due to the more rapid dissolution of the amorphous drug molecules compared to the crystalline forms. PVP-4-HPR ASD was capable of maintaining a 1000-fold solubility enhancement (>300 µg/mL) over 7 days, while the in vivo release in rats from the PLGA PVP-4HPR-TEAC implants after subcutaneous implantations demonstrated a complete and continuous drug release (90%) for over 1 month.

A non-aqueous microemulsion formulation capable of forming a depot for the prolonged release of 4-HPR within breast tissue was designed as a preventive therapy for breast cancer [92]. Generally, a microemulsion is an isotropic dispersion of two non-miscible liquids in combination with a surfactant. Here, the ingredients of the formulation included phosphatidylcholine/tricaprylin/propylene glycol in the ratio 45/5/50 (*w*/*w*/*w*). Upon water uptake, the microemulsion released in vitro 30% of 4-HPR after 9 days. This slow release might result from the high affinity of the drug to the ingredients. Consistent with the slow delivery, the formulation formed a depot in vivo with a prolonged fluorochrome release for 30 days without producing any signs of local irritation. In a model of chemically induced breast cancer in female rats, a dose of 50 mg 4-HPR was given every 3 weeks for 3 months. The microemulsion significantly reduced (4.7-fold) the incidence of breast tumors but no differences in the tumor volumes among the groups were detected. Drug levels in the plasma of animals treated with the 4-HPR-loaded microemulsion were below the detection limit of the method, while an average of 1.3 μg/mL of the drug was detected in the mammary tissue of treated animals after 30 days. These results support previous evidence that the drug can accumulate in breast tissue and the potential applicability of this strategy to local chemoprevention.

## 4. Future Perspectives and Conclusions

Early clinical trials revealed that the capsular 4-HPR formulation demonstrated limited evidence of activity even at high doses, with mean drug plasma levels ranging from 6 to 13 µM. The interpatient variability in the outcomes was mainly ascribed to the hepatic first-pass effect, thus establishing that the maintenance of therapeutic drug levels strongly depends on the patient’s metabolism rate, which may be fast or slow. In addition, the absorption of the drug, which is regulated by its dissolution rate, is very slow as 4-HPR is insoluble in water. These considerations mean the parenteral route of administration seems almost obligatory in order to overcome these unfavorable biopharmaceutical features, despite being not the first choice in the administration of a medication formulation due to reduced patient compliance. Indeed, it has been found that in the case of 4-HPR-hydroxypropyl β-cyclodextrin complex, when the same formulation was administered both orally and intravenously at the same concentration, high drug plasma levels were warranted only in the latter case (C_max_ = 6932 ng/mL and 730 ng/mL, i.v and per os, respectively). Moreover, this formulation could be toxicologically safer and regarded more favorably in comparison to lipid-based formulations where hypertriglyceridemia and prolonged infusion times can lead to a reduction in the dose and to less adherence to the therapy, as reported in the clinical phase I trial [88]. Recently, the FDA published a list of 23 newly accepted cyclodextrin-based formulations, of which 9 are based on HPβ-CD. As a confirmation of the safety of the ingredients, to date, marketed products are present for all routes of administration [93]. Finally, the paradigm shift towards intravenous formulations is evidenced by the higher number of research studies performed. To date, the number of parenteral formulations tested in clinical and preclinical studies is almost double that of oral studies.

Currently, the efforts of the scientific community aimed at supporting the relevance of 4-HPR can be divided into two research areas. One deals with the synthesis of new retinoid analogs, while the other concerns the search for new drug delivery systems. Regarding the designing of new retinoids, 9-cis-UAB30 (8-(3,4-dihydro-1′(2′H)-naphthalen-1′-ylidene)-3,7-dimethyl-2,4,6-octatrienoic acid, UAB30) seems to be the most promising molecule. It is a novel selective RXR agonist that has demonstrated similar efficacy but a minimal toxicity profile when compared to ATRA [94]. It has demonstrated a favorable pharmacological profile with a half-life longer than that of isotretinoin. Additionally, it has proven effectiveness in preclinical breast cancer prevention and in xenograft models of neuroblastoma [94].

Concerning the development of new drug delivery systems to improve the bioavailability and pharmacokinetic profile of existing lipophilic drugs found active in vitro, several strategies have been attempted. As abovementioned, lipid matrix, oil in water (o/w) emulsions, ion-pair complexes, inclusion complexes, micelles, polymeric micelles, conjugated forms, liposomes, nanocapsules, and microspheres were developed. In any case, while the most common formulation typologies were pursued, the use of endogenous carriers like extracellular vesicles (EVs), which have been already proposed for the loading of paclitaxel, another lipophilic anticancer drug, remains underexplored [95]. They could be an interesting vehicle suitable for cancer treatment thanks to their intrinsic properties of targeting and tissue homing, and because they are not immunogenic. However, in this case, the formulation should be administered intravenously because, as evidenced in this review, this route of administration is the most effective for 4-HPR. 4-HPR is a synthetic derivative of vitamin A (retinol) that belongs to the second generation of retinoids. Several in vitro and preclinical studies have demonstrated its promising anticancer activity in many types of cancer, both in adults and pediatrics. However, 4-HPR fails to reach the market due to the unsatisfactory results shown in clinical investigations, mostly attributable to its low bioavailability. In this regard, clinical studies have rarely reached or successfully passed Phase II, since its effectiveness as an anticancer drug was not fully demonstrated, although it has demonstrated an encouraging safety profile and hopeful outcomes in vitro. This lack of clinical efficacy is presumably due to the relatively low plasma concentrations of 4-HPR, which were often variable and below the therapeutic threshold. These unreliable clinical results associated with the drug’s unpatentability have reduced the interest of pharmaceutical companies in 4-HPR. Nevertheless, researchers from academia and hospitals all around the world have been trying to keep the promise of 4-HPR alive by developing new 4-HPR formulations that claim to successfully address the bioavailability limitations.

## Figures and Tables

**Table 1:** Recognized retinoid generations.

Generation	Description	Compounds
First	Naturally occurring compounds and their isomers	Retinol, retinal, tretinoin *, isotretinoin, Alitretinoin
Second	Mono-aromatic synthetic analogs formulated exclusively for oral dosing	Etretinate, acitretin **, motretinide, Fenretinide
Third	Retinoidal polyaromatic compounds	Arotinoids, adapalene, bexarotene, Tazarotene, tamibarotene
Fourth	Pyranone derivatives for topical use with selectivity towards the RARs located in the epidermis	Trifarotene, seletinoid G

* Retinoic acid; ** metabolite of etretinate; RAR = retinoic acid receptors.

**Table 2:** Retinoids approved or in clinical trials indicated for cancer treatment.

Compound	Agency	Year	Indication	Actual Use in Cancer	Ref.
ATRA	FDA	1995	APL	As approval	[53]
	EMA				
13-cis retinoic acid	FDA	1982	Severe acne	Neuroblastoma	[54]
	EMA	2003			
Bexarotene	FDA	1999	Refractory cases of cutaneous T-cell lymphoma	As approval	[55]
	EMA	2001			
4-HPR	In clinical trials			Neuroblastoma	[56]
Tamibarotene				Neuroblastoma	[57]

FDA = Food and Drug Administration; EMA = European Medicine Agency; APL = acute promyelocytic leukemia.

**Table 3:** Pediatric and adult cancer trials for existing malignancies employing 4-HPR as a single agent.

Indication	Sponsor	Phase	Formulation	Daily Dose	Enrolment	PL ^a^ (μmol/L)	Response	Ref.
Prostate cancer	CTRG	II	Capsule	1800 mg	27	^b^	≥50% reduction in PSA in 1/27	[66]
Prostate cancer	CCC	II	Capsule	1800 mg	23	^b^	No objective response	[67]
Brain tumors	NABTC	II	Capsule	1600–1800 mg	45	2 ± 0.9	No activity	[68]
Ovarian cancer	CCC	II	Capsule	1800 mg	31	12.5	OS at 18 mo 66% in >9 µmol/L	[69]
Solid tumors	BAKCI	I	Capsule	500–4800 mg/m^2^	31	Unknown	No activity	[70]
Renal cell carcinoma	BAKCI	II	Capsule	1800 mg/m^2^	19	^b^	No activity	[60]
Small cell lung cancer	UMCC	II	Capsule	1800 mg/m^2^	19	7 ± 4	No objective responses	[71]
Pediatric neuroblastoma	IG & INT	I	Capsule	100–4000 mg/m^2^	54	13 ± 6 ^c^	No CR/PR, 41 SD	[8]
Pediatric solid tumors	COG	I	Capsule	350–3300 mg/m^2^	54	10 ± 3	1 CR, 13 SD	[61]
Pediatric neuroblastoma	COG	II	Capsule	2475/1800 mg/m^2^ fixed	58	8 ± 3	1 PR, 7 SD	[63]
Pediatric neuroblastoma	NANT	I	4-HPR/LXS	352–2210 mg/m^2^	32	16 ± 3	4 CR, 6 SD	[56]
Pediatric neuroblastoma	NANT	I	4-HPR/LXS + ketoconazole	1500 mg/m^2^ fixed	N.R.	18 ± 4	On-going	[72]
Hematologic malignancies	NCI	I	4-HPR-ILE	80–1810 mg/m^2^	19	> 50 µmol/L	36% CR + PR	[73]

PL = plasma levels; IG = Istituto Gaslini; INT = Istituto Nazionale Tumori; COG = Children’s Oncology Group; BAKCI = Barbara Ann Karmanos Cancer Institute; UMCC = University of Michigan Cancer Center; NCI = National Cancer Institute; EIO = European Institute of Oncology; CCC = California Cancer Consortium; CTRG = Cancer Therapeutics Research Group; NABTC = North American Brain Tumor Consortium; NANT = New Approaches to Neuroblastoma Therapy Consortium; 4-HPR/LXS = oral formulation of 4-HPR in an organized lipid matrix (LYM-X-SORB); 4-HPR-ILE = intravenous formulation of 4-HPR in an intralipid emulsion; PSA = prostate-specific antigen; OS = overall survival; SD = stable disease; CR = complete response; PR = partial response. ^a^ Average C_max_ achieved at the highest dose administered. ^b^ No plasma PK is assessed. ^c^ The PK values are for day 28 of continuous 28-day dosing; N.R. = not reported.

**Table 4:** Oral formulations of 4-HPR developed and evaluated in vivo so far.

Formulation	Ingredients	Study Type	Treatment Plan	Results	Other	Ref.
Soft gel capsules	Corn oil TWEEN 80	Phase II NB	2475 mg/m^2^/d or 1800 mg/m^2^/d 7 days every 21 days Max 30 cycles	C_max_ = 6–13 µM	⬆ Tolerability ⬇ Compliance Interpatient variability	[63]
4-HPR LYM-X-SORB Lipid matrix	LPCH MG FFA	Preclinical	PK study 120–360 mg/kg/d twice a day 9 doses	C_max_ > 3-fold vs capsular 4-HPR in mice	⬇ Toxicity Interpatient variability ⬇ Tolerability in adults GIT irritation	[56,76,77]
		Phase I NB	352 mg to 2210 mg 4-HPR/m^2^/day BID, 7 days, every 3 weeks	C_max_ = 21 µM		
		Phase I solid tumors	1000 mg/m^2^/day TID, 7 days, every 3 weeks	MTD = 800 mg/m^2^/day		
4-HPR Nanocapsules	GB GEL	Preclinical NB SC model	Efficacy study: 200 mg/kg, 3 days /week for 3 weeks Adsorption study: 100 mg/kg, 5 days	C_max_ = 6 µM ITC = 12 µM ⬇ Tumor growth in fasting conditions	Mean size = 213 mm EE% = 87% DL% = 12%	[78]
PVP 4-HPR Spray-dried Amorphous solid dispersion	PVP	Preclinical	PK study SD (20 mg/kg)	AUC_0–48_ = 5911 ng × h/mL C_max_ = 505 ng/mL	⬆ Bioavailability Storage stability at 5 °C and 60% RH for up to 6 months	[79]
NanoFEN Inclusion complex	2-HP-βCD	Preclinical	PK study SD (5 mg/kg)	C_max_ = 730 ng/mL (2-fold ⬆ vs. capsular 4-HPR) AUC_0–last_ = 9378 h × mg/mL (3-fold ⬆ vs. capsular 4-HPR)	⬆ Solubility by 1409-fold ⬆ Anticancer activity in vitro	[80]
Bio-nFeR Micelles	PCH GTB	Preclinical Lung Melanoma Colon SC model	PK study SD =1,050,100 or 200 mg/kg CA = 150 mg/kg, 5 days on and 2 days off for 3 weeks	SD (200 mg/kg): C_max_ = 9.2 µM AUC_0–last_ = 112,957 ng × h/mL CA: C_max_ = 12 µM AUC_inf_ = 85,378 ng × h/mL ITC = 5 μM	Mean size = 300 nm EE% = 92% DL% = 9% 300 mg/mL Bio-nFeR provide 25 mg/mL 4-HPR in solution	[81]

PVP = polyvinylpyrrolidone; LPCH = Lysophosphatidylcholine; PCH = phosphatidylcholine; GB = glucosamine butyrate; GEL = gelatine; GTB = glyceryl tributyrate; MG = monoglycerides; FFA = free fatty acids; PK = pharmacokinetic; SD = single dose; BID = divided into two doses daily; TID = divided into three doses daily; EE = Encapsulation Efficiency; DL = Drug Loading; MTD = maximum tolerated dose; 2-HP-βCD = 2-hydroxypropyl β-cyclodextrin; CA = chronic administration; ITC = intratumor drug concentration; SC = subcutaneous; RH = relative humidity; ⬆ high, higher, improved, increased; ⬇ low, lower, decreased.

**Table 5:** Parenteral formulations of 4-HPR developed and evaluated in vivo so far.

Formulation	Ingredients	Study Type	Treatment Plan	Results	Other	Ref.
4-HPR-PVA Polymeric micelles	PVA	Preclinical Metastatic NB	Efficacy study 13.5 mg/mL, 5 times, every 3 days (i.v.)	⬆ Solubility by 200-fold ⬆ MST	Mean size = 350 nm Constant in vitro release	[82]
4-HPR Bz-PEG-PAS Polymeric micelles	PEG-cPA-Bz	Preclinical Melanoma SC model	PK study SD (75 mg/kg, i.v.) Efficacy study 75 mg/kg, 3 times every 2 days (i.v.)	⬆ Residence time ITC = 56.6 μg/g after 6 h	EE% = 70% Mean size = 173 nm	[83]
Liposomal 4-HPR	HSPC, CHE DSPE-PEG2000 NGR *	Preclinical Orthotopic NB	Efficacy study 1 mg/kg twice a week, 6 weeks (i.v.)	Vascular targeting ⬆ Efficacy	Mean size = 142 nm EE% = 69% ⬇ Drug leakage	[84]
4-HPR HSA Nanocapsules	HSA	Preclinical Lung adenocarcinoma SC model	Efficacy study 1 mg/kg, 12 times every 3 days (i.v.)	⬇ 63% tumor volume vs. CTR ITC = 5.7 µM ⬆ Bioavailability	Size = 80–100 nm DL% = 14%	[85,86]
4-HPR PLGA Microspheres	PLGA Brij 98 MgCO_3_	Preclinical	PK study SD (4.2 mg, i.m.)	C_max_ = 0.24 µM on 10th day 101% Bioavailability T_max_ = 240 h	Size = 5–10 µm Sustained release	[87]
20% 4-HPR-soy O/W	Soy oil Egg phospholipids	Phase I Hematologic malignancies	80–1810 mg/m^2^/d, on days 1 to 5, 21-day cycles (c.i.v.)	MTD = 1280 mg/m^2^/d for 5 days ⬆ C_max_ 5-7-fold vs capsular 4-HPR	Dose-limiting toxicity ⬆ Time drug infusion ⬇ Potency For T-cell lymphomas	[73]
		Phase I Solid tumors	1280 mg/m^2^/d for 5 consecutive days in a 21-day cycle (c.i.v.)	Dose reduction needed C_max_ = 9.9–10.8 µg/mL	⬆ Adverse events ⬇ Activity	[88]
NanoFEN Inclusion complex	2-HP-βCD	Preclinical Lung Colorectal SC model	PK study Single dose (5 mg/kg, i.v.) Efficacy study 10 mg/kg three times/week (i.p.)	C_max_ = 6932 ng/mL AUC_inf_ = 13,657 ng × h/mL Relapse prevention	⬆ Efficacy in vitro ⬇ Tumor mass	[80]
Fen-RC16+ Micelles	C16-ceramide^+^	Preclinical NB SC model	Efficacy study 1.02 mg/kg 8 times every other day (i.v.)	⬇ Tumor mass Relapses prevention	Size = 20–40 nm Solubility = 1.5 mg/mL ⬆ Intracellular uptake	[89]

PK = pharmacokinetic; SD = single dose; SC = subcutaneous; NB = neuroblastoma; PEG-cPA-Bz = Poly-(ethylene glycol)–poly(aspartate) block copolymer with benzyl groups; O/W = oil in water emulsion; MST = mean survival time; PVA = polyvinyl alcohol; Bz-PEG-PAS = poly-(ethylene glycol)–poly(aspartate) block copolymer modified with benzyl groups; * active targeting peptide directed to tumor endothelial cells; ITC = intratumor concentration; HSPC= hydrogenated soy phosphatidylcholine; CHE = cholesterol; DSPE-PEG2000 = 1,2-distearoyl-glycero-3-phosphoethanolamine-N-[methoxy(polyethyleneglycol)-2000]; EE = encapsulation efficiency; DL = drug loading; HAS = human serum albumin; PLGA = poly-lactic and glycolic acids; 2-HP-βCD = 2-hydroxypropyl β-cyclodextrin; CTR = control; MTD = maximum tolerated dose; i.v.= intravenous; i.m. = intramuscular; c.i.v. = continuous intravenous infusion; i.p. = intraperitoneal; ⬆ high, higher, improved, increased; ⬇ low, lower, decreased.

## Data Availability

No new data were created or analyzed in this study. Data sharing is not applicable to this article.
